# Isolation and Characterization of Nocardiae Associated with Foaming Coastal Marine Waters

**DOI:** 10.3390/pathogens10050579

**Published:** 2021-05-10

**Authors:** Luke Wright, Mohammad Katouli, D. İpek Kurtböke

**Affiliations:** Genecology Research Centre, School of Science, Technology and Engineering, University of the Sunshine Coast, Maroochydore, QLD 4558, Australia; Luke.Wright@research.usc.edu.au (L.W.); mkatouli@usc.edu.au (M.K.)

**Keywords:** *Nocardia*, Actinobacteria, pathogenicity, foaming coastal waters, antibiotic resistance, *Nocardia*-phage

## Abstract

Nocardiosis is an infectious disease caused by *Nocardia* species that occurs worldwide, albeit more prevalently in tropical/subtropical regions. It can appear as either acute, subacute or as a chronic infection mostly with those with a compromised/weakened immune system. Inhalation of spores and or mycelium fragments is the main transmission route for developing pulmonary nocardiosis. In contrast, cutaneous nocardiosis usually occurs via direct contact. In the subtropical region of the Sunshine Coast in Australia foaming events with thick and persistent and orange-brown color foam have been observed during summer seasons in the near shore marine environments. This study reports the existence of nocardiae in these near shore marine environments by the use of a novel isolation method which used the gas requirements of nocardiae as a selective battery. A total of 32 nocardiae were isolated with the use of this novel method and subsequently conducted molecular identification methods confirmed that the isolates belonged to the genus *Nocardia*. Twenty-one isolates out of the 32 were closely related to *N. nova* strains MGA115 and one was related to CBU 09/875, in addition when compared with human pathogenic nocardiae twenty of the isolates were found to be related to *N. nova* strain JCM 6044. Isolates displayed varied resistance against some of the antibiotics tested when interpretation threshold recommended the Comite de L’Antibiogramme de la Societe Francaise de Microbiologie were used. The highest level of resistance against cefotaxime (*n* = 27) and ceftriaxone (*n* = 24). Some of the isolates (*n* = 6) that displayed resistance to selected antibiotics also possessed potential human pathogenic characteristics such as adherence and translocation through human long epithelial cells as well as displaying phage resistance (*n* = 26). They might thus present a potential public health risk if frequently encountered through exposure to aerosols generated by the foam as well as direct contact through a wound. Preventative measures to control the growth of nocardiae in such environments such as the control of pollutants, might prevent potential infections that might be caused by these bacteria in humans as well as in marine animals.

## 1. Introduction

Members belonging to the genus *Nocardia* of the phylum Actinobacteria and the order *Corynebacteriales* [1] are classified as Gram-positive (although many species display a level of acid fastness) and filamentous bacteria [2]. They are widely distributed [3,4,5], mostly saprophytic and ubiquitous in the environment and commonly found in soil, water bodies, and decaying vegetation [6]. One source where nocardiae are most predominant is wastewater recycling plants, specifically, in aerated activated sludge treatments [7,8,9,10]. The wastewater recycling industry normally utilizes nocardiae and other filamentous microorganisms for bioremediation in the anoxic phase of their treatment plants to metabolize dissolved nitrates (NO_3_^-^) [7,11,12]. However, if the denitrifying process in the anoxic phase is inadequate, brownish colored foam/sludge may form on the surface of the final clarifier [13].

Some of the foaming events in aquatic environments have been linked to the presence of pollutants [14]. Similarly, the foaming events in the Sunshine Coast region of Australia along coastal shorelines, lakes and streams were frequently coincided with adverse/turbulent weather conditions when excess pollution in effluent water runoff at near-shore marine environments was observed https://www.qld.gov.au/environment/pollution/management/disasters/flood-impacts They carried similar characteristics of the foam color and density reported by Fryer and Gray [15]. Kurtböke [16,17,18] reported the existence of nocardiae in the foaming coastal marine waters as identified via the use of molecular methods. Some of the isolates were closely related to human pathogenic ones reported in other parts of the world [17].

Although, species belonging to the *Nocardia* cluster causing human diseases are generally considered opportunistic pathogens, nocardiosis is reported to manifest in immuno-competent people and according to the Genetic and Rare Diseases Information Center [19] approximately one third of infected individuals are immune-competent. Although the number of known *Nocardia* species responsible for disease in humans is also increasing [20], a review of the literature on nocardiosis in humans by Kandi [21] emphasized otherwise. One of the reasons for this fact might be due to the lack of epidemiological data on the incidence of nocardiosis which may not be truly reflective of the pathogenic potential of *Nocardia* species. This might be partly due to the fastidious growth patterns of certain species of *Nocardia* or lack of rapid diagnostic tests required for their detection and subsequent isolation in a clinical laboratory [22].

Numerous studies to date have identified three main virulence factors that pathogenic *Nocardia* species may use to overcome host’s immune system and cause disease [23,24,25]. These factors include the presence of trehalose 6-6’-dimycolate (TDM), superoxidase dismutase and the well-categorized antioxidant enzyme catalase. TDM is a glycolipid molecule found in the cell walls of numerous *Nocardia* species. TDM is shown to reduce and inhibit the phagocytic process of the host’s immune cells e.g., macrophages. It has been suggested that the trehalose levels in microorganisms may vary considerably depending on the environmental conditions, and higher levels of trehalose may increase the virulence potential of a microorganism [26]. Superoxidase dismutase and catalase are potent antioxidant enzymes that offer a level of resistance against oxidative enzymes produced by foreign or host cells. 

Adhesion, invasion and translocation assays in vitro using human cell lines are typically used to assess and quantify the protective capability of cells against a pathogenic organism as well as the ability of the invading organism to adhere and navigate the host’s immune defense. Whilst some studies have focused on using murine and human cell lines to assess the adhesion and translocation properties of pathogenic *Nocardia* species [27,28,29], cultured strains isolated from diseased human tissue were used in the tests. Hence, little is known about these pathogenic characteristics of *Nocardia* species isolated from environmental samples; particularly from coastal foaming marine waters and sandy beaches that are commonly used for recreational activities. 

In the view of the above, this study was undertaken to determine the presence of nocardiae in foaming near shore marine waters with use of a novel selective isolation method. Isolated nocardiae was subsequently investigated for their pathogenic properties using Calu-3 cells (ATCC HTB-55) and their antibiotic and phage susceptibility patterns were also determined. 

## 2. Results

### 2.1. Selective Isolation and Molecular Level Identification of Nocardiae

**Figure 1:**
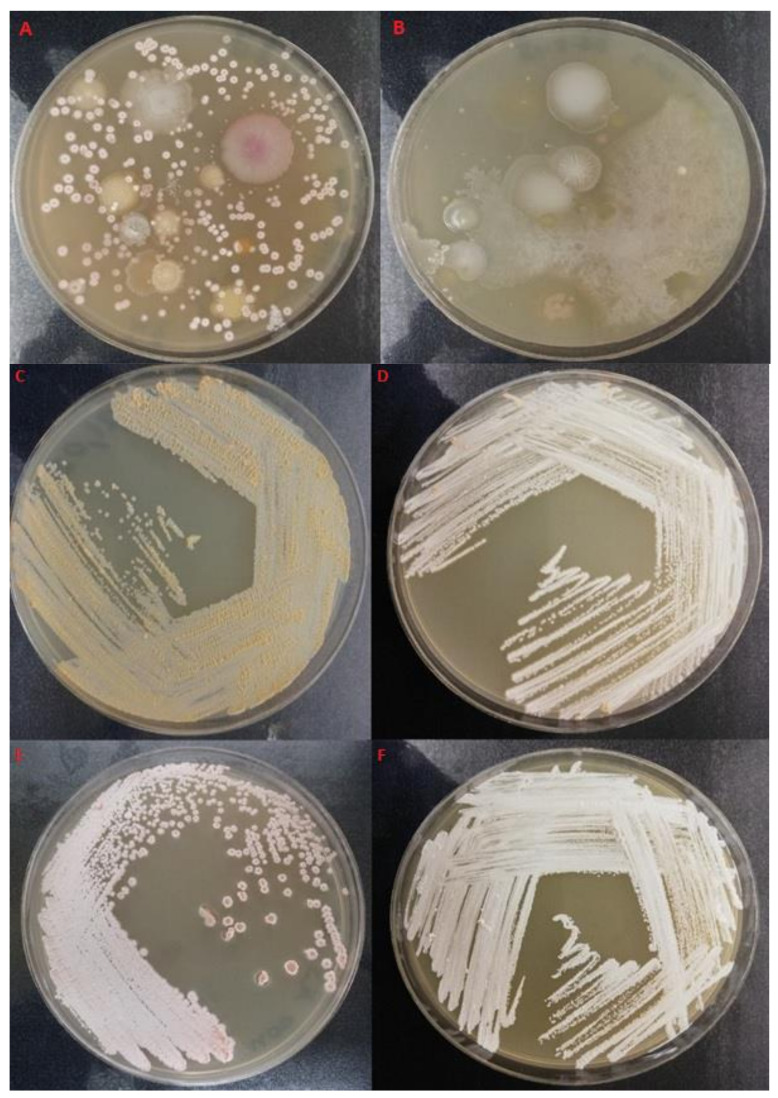
Isolation of nocardiae via exposure to anaerobic (**A**) and anaerobic (**B**) conditions first and then incubating them under aerobic conditions. Typical white/pinkish/salmon colored colonies of *Nocardia* isolates from the coastal foaming marine waters on GYM-*Streptomyces* medium: (**C**) USC-21021, (**D**) USC-21044, (**E**) USC-21038 and (**F**) USC-21046.

The novel selective isolation method employed resulted in the removal of background microbiota and successful isolation of *Nocardia* species. Facultative anaerobic characteristics of nocardiae resulted in their survival in the anaerobic chamber and clearance of background bacterial flora (Figure 1 A–F) which would under aerobic conditions would overgrow nocardiae colonies and render them unculturable.

**Figure 2:**
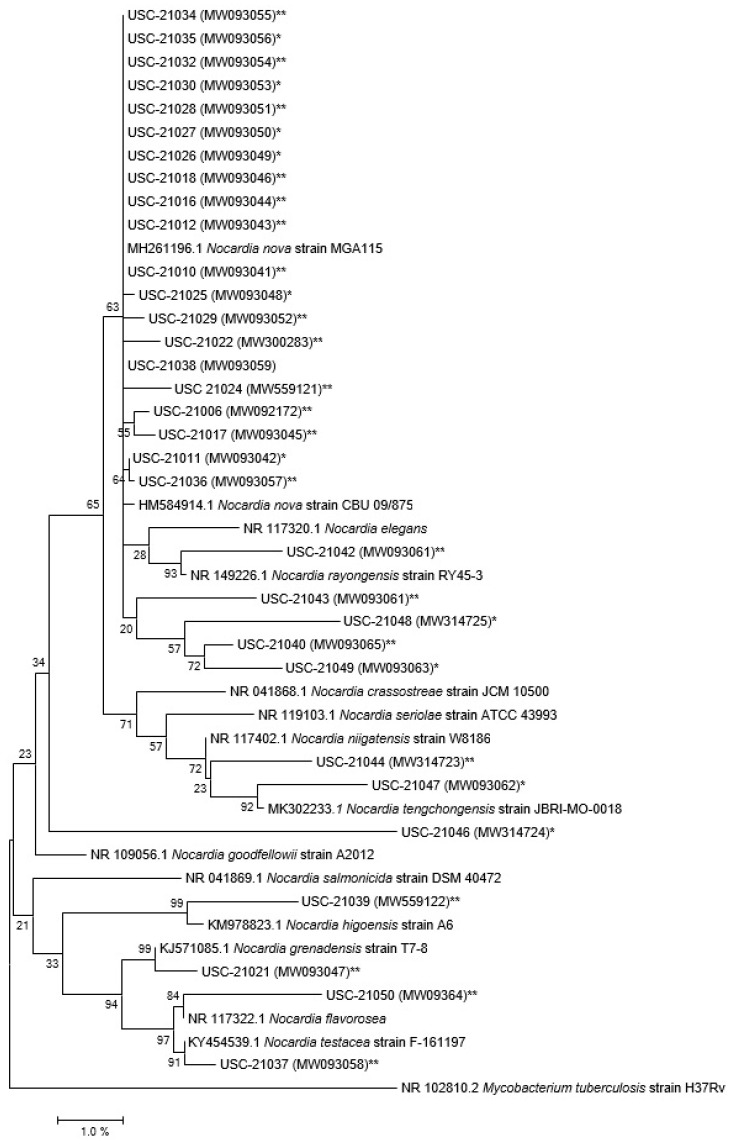
Phylogenetic tree of the 32 isolates using their 16s rRNA gene sequences in relation to their closest relatives. Bootstrap values (≥50%) are indicated at nodes. The scale bar represents percentage (%) divergence. The accession number of the closest relatives is included. *Mycobacterium tuberculosis* was chosen as an out-group sequence to root the tree. *: isolated using aerobic conditions, **: isolated by exposing anaerobic conditions first followed by incubation at aerobic conditions.

**Figure 3:**
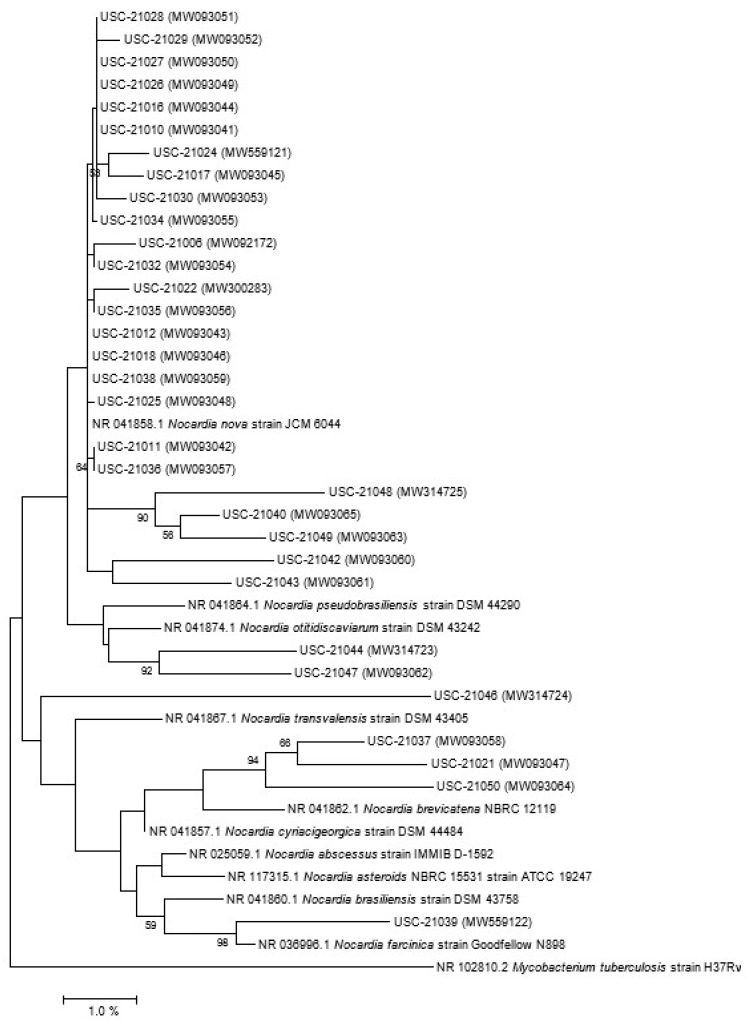
Phylogenetic tree of the *Nocardia* isolates using their 16s rRNA gene sequences in relation to their closest relatives identified as being clinically significant and grouped according to seven antimicrobial susceptibility patterns [30]. Bootstrap values (≥50%) are indicated at nodes. The scale bar represents percentage (%) divergence. The accession number of the closest relatives is included. *Mycobacterium tuberculosis* was chosen as an out-group sequence to root the tree.

When isolation plates were subjected to anaerobic conditions first 62.50% of nocardiae were cultured compared to the 37.5% when direct aerobic conditions were used. A total number of 32 confirmed *Nocardia* species were isolated following molecular level characterization (Figure 2, Table S1) and some of them were closely related to the pathogenic species of nocardiae (Figure 3).

Twenty-one isolates out of the 32 were closely related to *N. nova* strains MGA115 and one was related to CBU 09/875 (Figure 2), in addition when compared with human pathogenic nocardiae twenty of the isolates were found to be related to *N. nova* strain JCM 6044 (Figure 3).

**Figure 4:**
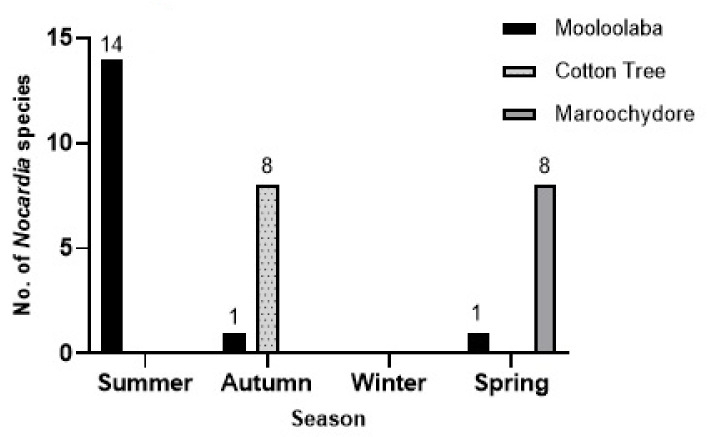
The total number of *Nocardia* species isolated during a 1-year period from the foaming marine waters of the Sunshine Coast region.

Nocardiae presence was greater in the summer months confirming the temperature dependent existence of the taxon members in the near-shore marine environments. In winter months no nocardiae were isolated when both aerobic and anaerobic isolation methods were used (Figure 4, Table S2).

### 2.2. Antibiotic Susceptibility Profiles of the Isolates

*Nocardia* isolates displayed resistance against some of the antibiotics tested with cefotaxime, and ceftriaxone showing the highest level of resistance (Table 1, Table S2).

### 2.3. Adhesion of Nocardia Isolates to Calu-3 Cells

The degree of adhesion of the *Nocardia* isolates (*n* = 32) to Calu-3 cells varied significantly (*p* *<* 0.0001) between the isolates, with six isolates showing the highest degree of adhesion per cell and had also colonised more than 50% of the cells (Table S3). Isolate USC-21034 displayed the highest degree of colonization and adhesion (>99%) per Calu-3 cell. The degree of adhesion for the positive control *E. coli* strain HLMN-1 was 21.8 ± 0.6 (90%) whereas for the USC-21034 isolate it was 15.5 ± 0.8 CFU/cell.

### 2.4. Translocation of Nocardia Isolates

The confluency and the integrity of the Calu-3 cells were initially tested by measuring the Transepithelial electrical resistance (TEER) value of the culture over 16 days. The results indicated that cells reach its maximum confluency after 10 days (Figure S1) and therefore all translocation experiments (maximum 2 h for each assay) were done on day 10 when the TEER value reached 1588 Ω cm^2^. 

**Figure 5:**
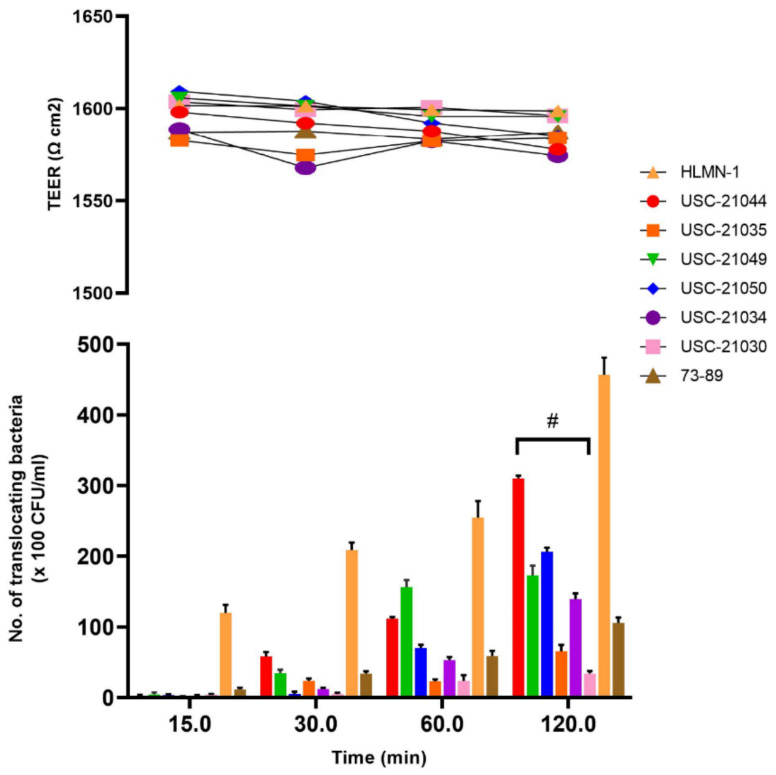
The rate of translocation of six selected nocardiae isolates over 120 min using Calu-3 cell line. *Escherichia coli* strains (HMLN-1 and 73–89) were used as positive and negative control respectively. Data shown are from triplicate experiments and plotted as the mean ± SEM. Transepithelial resistance remained stable throughout the test.

The six selected *Nocardia* isolates with the highest degree of adhesion per cell as well as the highest degree of colonization were tested for their rate of translocation via Calu-3 cells. The overall translocation ability of the isolate USC-21044 was significantly greater (*p* = 0.0164) compared to the other five *Nocardia* isolates difference in degrees of translocation of the *Nocardia* isolates over the 120 min assay compared to the controls was also significant (*p* = 0.054) (Figure 5). During the translocation assays TEER values remained constant indicating that the translocation occurred via Calu-3 cells and not between the cells (Figure 5).

### 2.5. Detection of Nocardia-Specific Phage Susceptibility of the Isolates

Majority of the nocardiae isolates were found to be resistant to the nocardiae-specific phages (Table 2).

## 3. Discussion

Investigative studies were conducted to determine whether nocardiae would be present in the foaming coastal marine waters increasingly encountered at the Sunshine Coast region of Australia [18,32,33] similar to the nocardiae regularly detected in activated sludge systems [8]. To determine the presence of the species of the genus *Nocardia* in the foam samples a novel selective isolation method was used. The selective battery used stemmed from the exploitation of the gas requirements of nocardiae during their laboratory growth. The novel method developed allowed successful isolation of nocardiae without background contamination thus further facilitating their transfer onto purification media. When anaerobic conditions are used 62.50% of nocardiae were cultured compared to the 37.5% when aerobic conditions were used, confirming the effectivity of the novel method developed in the study. Selective isolation of microorganisms is of significant value as it generates information on the morphologies and growth patterns of microorganisms under laboratory conditions [18,32,33]. The successful isolation of nocardiae in this study thus furthered information on the in vitro characteristics of the local isolates which are of importance in terms of definition of chemotherapy regimens in the event of local infections. Moreover, laboratory studies on the cultured representatives of nocardiae can provide further information such as the detection of pathogenicity and hydrolytic capabilities of the organisms. Such information can further be used for pollution control in coastal marine waters where nocardiae with such characteristics can survive and persist [34]. In addition, sewage outflows during cyclonic events can introduce human pathogenic nocardiae into recreational environments such as local beaches [35]. *N. farcinica* as an example has been reported to be an opportunistic pathogen in activated sludge plants [36]. Vautrin et al. [35] have recently indicated that urban infiltration basins can accumulate urban pollutants and favor the growth of potentially pathogenic biological agents as *Nocardia*.

The majority of the nocardiae isolates were also found to be resistant to the phages in the study. Since these phages were originally isolated using type strains of non-Australian origin, the resistance observed by the isolates might indicate endemicity for some of the local isolates. If bacteriophages are used to control nocardia infections data like the one obtained in this study can provide background information when phage cocktails are designed for therapy purposes which are of importance for the acceptance and implementation of phage therapy [37,38].

Over 70% of resistance was detected for cefotaxime and ceftriaxone among the local isolates when interpretation threshold recommended the Comite de L’Antibiogramme de la Societe Francaise de Microbiologie were used [39,40]. Currently, there are different tests used to determine nocardiae antibiotic susceptibility patterns. These tests are (i) disk diffusion, (ii) broth dilution, (iii) agar dilution, (iv) epsilometer test (E-test) and (v) the BACTEC radiometric method. Recent study by Lebaux et al. [39] concludes that the Antibiotic Susceptibility Test (AST) performed by disk diffusion method appears to be a reliable method for routine testing of *Nocardia*. They, however, recommend that for trimethoprim-sulfamethoxazole, if the inhibition zone is <10 mm, as in our findings (Table S2), an E-test strip should be performed. 

A recent study by Tan et al. [41] highlighted the importance of routine antimicrobial susceptibility testing of *Nocardia* as a result of detection of sulfonamide resistance and atypical antibiograms obtained in their laboratory in Sydney, Australia. Although the percentage of *N. nova* they reported (80/270) was lower than our study (22/32) our result on amikacin susceptibility also agreed with Tan et al. [41] investigation that found amikacin as one of the most effective antibiotics tested in their laboratory against nocardiae. All these findings again stress the importance of uninterrupted wide scale surveys for the detection of pathogenic bacteria for disease prevention and design and implementation of prophylactic measures.

There is still limited information on the pathogenicity of non-clinical but environmental *Nocardia* species regarding their adhesion and translocating abilities on the human cells. Moreover, no studies to date have correlated a link between coastal marine water foaming events and an increase in human pulmonary and or cutaneous nocardiosis cases despite the fact that pathogenic *Nocardia* species are known to become aerosolised and infect a human host [42]. The primary site of infection for *nocardiosis* to manifest in humans is in the lungs via inhalation of *Nocardia* cells and spores, and with direct skin contact; a primary site that has the potential to spread to other parts of the body (disseminated nocardiosis). In this study, *Nocardia* species isolated from beach foam were found to adhere and translocate with a varying degree of efficiency in Calu-3 cells *in vitro*. Findings are of significance for contact tracing if disease occurs as well as informing swimmers about the health risks of nocardiae who may be exposed to such species during shoreline foaming events. 

From the six isolates chosen for the translocation assay, two isolates (USC-21044 and USC-21050) displayed a reasonably high level of translocation. Isolate USC-21044’s closest relative is *Nocardia niigatensis and* there is little to no evidence in the scientific literature of *Nocardia niigatensis* causing pulmonary nocardiosis in humans. However, pulmonary nocardiosis is known to mimic pulmonary tuberculosis in clinical manifestations, such as in its signs and symptoms, radiological and laboratory results [43]. Hence, pulmonary nocardiosis has the potential to be misdiagnosed as pulmonary tuberculosis or *vice versa*. A recent study by Muricy et al. [43] reported isolating nocardiae from sputum samples from patients that were initially thought to be suffering from an infection caused by *Mycobacterium tuberculosis.* Isolate USC-21050’s closest relative is *Nocardia flavorosea* and is not again a typically isolated *Nocardia* species from patients suffering nocardiosis. Although, a study by Tan et al. [44] after having reviewed medical records from the National Taiwan University Hospital (between 1998–2008), noted that *Nocardia flavorosea* had been isolated from four patients suffering from nocardiosis.

Interestingly, numerous isolates in the study whose closest relative was identified as being a *Nocardia nova* species/strain displayed significant less adhesion and ultimately less translocation potential in contrast to the other two isolates USC-21044 and USC-21050. There is, however, little to no data available in the scientific literature on the adhesion, and translocation capabilities of pathogenic *Nocardia* species associated with coastal foaming marine waters using a human cell lung culture model in vitro. 

According to the Public Health Agency of Canada [45] the infectious dose (ID) and the incubation period of pathogenic *Nocardia* species in human hosts is unknown. Accordingly, data like the one presented in this study might be of importance indicting infectious capacities of locally isolated nocardiae in human hosts. Terrigenous origin actinomycetes can adapt to marine environments with diversified functions and adaptability [46] and increasing presence of fish pathogenic nocardiae in marine and aquaculture environments reported in other parts of the world might be an indication of such adaptation [47].

A review of the literature on nocardiosis in humans by Kandi [21] emphasized the lack of epidemiological data on the incidence of nocardiosis, and how this lack of data may not be truly reflective of the pathogenic potential of *Nocardia* species. This might be due to lack of sensitive identification tests and or the fastidious growth conditions certain species of *Nocardia* require for cultivation, isolation [48] and identification [49] in a clinical laboratory. However, as recently indicated by Mehta and Shamoo [22] advancements in rapid molecular diagnostic technology will soon place nocardiae in the “extended pantheon of medically important pathogens”. Accordingly, studies like the one presented here will generate further information on the occurrence and diversity of nocardiae in different environments and might have significance in linking the clinical data to the presence of disease causative species in the environment by the public health authorities.

## 4. Materials and Methods

### 4.1. Sampling Sites and Sample Types

Five locations along the Sunshine Coast beaches (1) Mooloolaba (26°40′58.6″ S 153°07′30.1″ E), (2) Maroochydore (26°39′17.7″ S 153°06′13.6″ E), (3) Cotton Tree (26°39′10.1″ S 153°05′56.9″ E), (4) Currimundi (26°46′03.8″ S 153°08′12.9″ E) and (5) Warana (26°43′09.9″ S 153°08′07.7″ E) were selected for foam sampling. These beach locations were selected due to their popularity with swimmers, varying beach conditions and history of foam formation. All beach locations were sampled over a one-year period during the spring, summer, autumn and winter to allow for a greater assessment of the nocardiae occurrence and diversity in near shore-foaming marine waters of this region in Australia.

**Figure 6:**
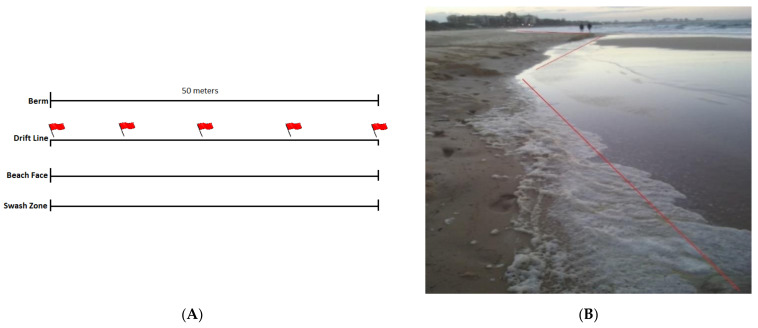
(**A**) Foam sampling design and (**B**) an example site of sampling along the drift line.

A 50 m stretch along the drift line of each beach location at 10 m intervals/transects was selected for foam sampling (Figure 6A,B). The samples were collected on the surface of the drift line and collected in 60 mL sterile specimen containers and stored on ice before being transported to the University of the Sunshine Coast (USC) for processing within 4 h of collection.

### 4.2. Isolation of Nocardiae Using a Novel Isolation Technique

Each foam sample (1 mL) was serially diluted and the aliquots of 0.2 mL from each dilution were transferred and spread onto six duplicates of the DSMZ medium #65 (GYM-*Streptomyces* medium [50] containing 50 ppm of cycloheximide and nystatin respectively to remove fungal contaminants, and allowed to dry for 20 min. One set of duplicate plates were pre-treated in an anoxic (CO_2_) environment (OXOID, Melbourne, Australia) at 28 °C for 48 h prior to being incubated and subsequently cultured at 28 °C in an O_2_ environment (standard atmospheric conditions). The remaining duplicates were directly incubated at 28 °C in an O_2_ environment without being pre-treated with CO_2_. Following ~2–3 weeks of incubation, colonies were selected based on typical *Nocardia* morphology as described in the Wink compendium [51].

Each selected isolate was then purified using DSMZ medium #65, and the plates were incubated at 28 °C in an O_2_ environment for ~2–3 weeks. Subsequently, following observable *Nocardia* morphology and nil signs of contamination, the isolates were cryopreserved in tryptic soy broth (TSB, OXOID) supplemented with 20% glycerol and stored at −20 °C prior to undergoing molecular identification.

### 4.3. Molecular Characterization of the Isolates

Direct colony polymerase chain reaction (PCR) adapted from Gathogo et al. [52] was used to extract the chromosomal DNA and amplify the 16s rRNA gene from the *Nocardia* isolates. The complete 16S rRNA gene was amplified using the HotStarTaq^®^ Multiplex PCR Kit (QIAGEN, Melbourne, Australia) for the extension of all primers. The amplification of the 16S rRNA gene was obtained by using the bacterial specific and universal primers 27F (5′-AGAGTTTGATCCTGGCTCAG-3′) and 1492-R (5′GGTT ACCTTGTTACGACTT-3′) as per Madueno et al. [53]. The PCR setting consisted of initial chromosomal DNA extraction and denaturation step at 95 °C for 15 min, followed by 34 cycles of 94 °C for 30 s, 60 °C for 1.5 min, 72 °C for 1.5 min, and the final extension was programmed at 72 °C for 10 min and hold on completion at 12 °C. The amplified PCR products were gel electrophoresed at 90 v for 90 min on a 1.0% agarose (Molecular Grade, Bioline, Sydney, Australia) in 0.6 × TrisBase EDTA (TBE) stained with ethidium bromide and viewed under a UV source for the presence of a 1500 bp DNA product; the PCR products were sequenced at Macrogen in Korea [31].

Forward and reverse sequences in FASTA format (including chromatogram) of the 32 *Nocardia* isolates were imported into DNA Baser Assembler (Version 5.15.0.087) [54] for contiguous (contig) ambiguity correction and sequence assembly (de novo).

The 16s rRNA gene sequences (contigs) of the 32 isolates and reference strains were imported into MEGA6 [55] in FASTA format and aligned according to MUSCLE [56]; Reference strain contigs were imported from the National Center for Biotechnology Information [57].

The phylogenetic analysis/evolutionary history was inferred by using the maximum likelihood method based on the Tamura-Nei model [58]. Sequence alignment and evolutionary analyses were conducted in MEGA6 [55] and the phylogenetic tree was constructed accordingly.

### 4.4. Detection of Antibiotic Susceptibility Patterns of the Isolates

Antibiotic susceptibility of the isolates and type strains were tested according to the disc diffusion method as outlined by the Clinical and Laboratory Standards Institute (CLSI) [59]; *Staphylococcus aureus* ATCC^®^ 25923, and *Escherichia coli* ATCC^®^ 25922 (only for imipenem 10 µg) were used as controls [39,40]. The 32 isolates, and type strains used as controls were tested in duplicates against ten different antibiotics (OXOID) (ampicillin (AMP: 10 µg), ceftriaxone (CRO: 30 µg), cefotaxime (CTX: 30 µg), imipenem (IMP: 10 µg), amikacin (AK: 30 µg), minocycline (MH: 30 µg), sulphamethoxazole/trimethoprim (SXT: 1.25/23.75 µg), erythromycin (E: 15 µg) and tobramycin (TOB: 10 µg). Results were recorded according to the zone diameter interpretive standards recommended the Comite de L’Antibiogramme de la Societe Francaise de Microbiologie, Recommendations 2013 [39,40].

### 4.5. Calu-3 Cell Line

The Calu-3 cells (ATCC HTB-55) obtained from the American Type Culture Collection (ATCC, Manassas, VA, USA) were maintained at 37 °C and 5% CO_2_ in a 50 mL culture flask in Eagle’s Essential Medium (EMEM, ATC302003). The medium was supplemented with 15% (*v*/*v*) fetal bovine serum (FBS, Moregate Biotech, Brisbane, Australia), and 1% (*v*/*v*) penicillin-streptomycin. Media was sterile filtered through a Millipore Express PES membrane (0.22 µm). The cell culture media was monitored and changed every 48 h until cell growth reached 95% confluence (~2.5 × 106 cells) using a disposable hemocytometer (INCYTO-C-Chip, ProSciTech, Townsville, Australia).

The *Nocardia* isolates were grown in 10 mL of TSB (OXOID) in an incubator shaker for 8 days at 28 °C and at 165 rpm. Prior to the adhesion assay, the *Nocardia* suspensions were centrifuged at 3500 rpm for 10 min and the supernatant discarded. The pellets were re-suspended in PBS (pH 7.4) at adjusted to the desired cell density (1.0 × 10^9^ CFU mL^−1^, OD = 1 at 600 nm); cell count was also verified by a cell plate count). *E. coli* strain HMLN-1 a professional translocating strain isolated from a mesenteric lymph-node of a fetal case of pancreatitis [60] and 73–89, originally isolated from ceacal contents of a hemorrhaged rat [61] were used as positive and negative controls respectively. They were grown in 10 mL of TSB in an incubator shaker for 2 days at 37 °C and at 165 rpm. Prior to the assay, the suspensions of control strains were centrifuged at 3500 rpm for 10 min and the supernatant discarded. The pellets were re-suspended in PBS (pH 7.4) at the desired cell density (1.0 × 10^9^ CFU mL^−1^, OD = 1 at 600 nm; cell counts were also verified by a cell plate count.

### 4.6. Adhesion and Translocation Assays

The adhesion and translocation assays performed were adapted from Vollmerhausen et al. [62]. Calu-3 cells were seeded into 8 well chamber slides (Lab-Tek II chamber, Thermo Fisher Scientific, Melbourne, Australia) filled with 0.3 mL of growth medium supplemented with 15% (*v*/*v*) FBS and 1% (*v*/*v*) penicillin-streptomycin and inoculated with a 1.7 × 10^5^ mL cells in each of the 8 chambers. The cells were grown to 100% confluence and prior to the adhesion assay, the medium was removed, and each chamber was washed three times with phosphate-buffer saline (PBS) with pH 7.4, Antibiotic free medium was then added to each chamber. Furthermore, to obtain an accurate multiplicity of infection ratio (MOI) the average of three chambers cell count at 100% confluence was used. It was established that the mean number of cells at 100% confluence per chamber was 200,000 cells; this cell count was used to determine the number of bacterial cells required to achieve a 1:50 MOI [62].

Subsequently, 10 µL of bacterial suspension (1.0 × 10^9^ CFU mL, OD = 1 at 600 nm) was inoculated into each chamber and incubated for 90 min at 37 °C. Cells were then fixed with 95% ethanol (*v*/*v*) for 5 min and stained using Giemsa stain for a period of 30 min, and observed using a light microscopy. All tests were performed in triplicate.

Bacterial adhesion per cell was determined by counting the number of adhering bacteria per 25 randomly selected Calu-3 cells. The percentage of bacteria colonizing Calu-3 cells was determined by randomly counting 100 Calu-3 cells and the mean number of cells showing bacterial adhesion was calculated after three replicate counting. Isolates showing greater than 50% adhesion to Calu-3 cells were selected for the translocation assay. These isolates also showed to have a higher number of bacteria per cells.

### 4.7. Trans-Epithelial Electrical Resistance Measurements and Translocation Assay

TEER was initially measured during the growth of Calu-3 cells using a Millicell-ERS voltammeter (MERS00001, Millipore, Melbourne, Australia); over 16 post seeding days to establish the TEER value of a confluence growth of Calu-3 cells. The 24-well plate was pre-filled with EMEM containing 15% FBS and 1% (*v*/*v*) penicillin-streptomycin at 37 °C. TEER between the inner and outer wells was measured and calculated using the following equation: measured monolayer resistance—measured blank resistance (filter without cells) × area of the filter (0.6 cm^2^) = trans-monolayer resistance (Ω cm^−2^) [63,64].

For the translocation assay, the Calu-3 Cells were seeded at 5 × 10^4^ cells into a 0.8 µm insert (12 mm diameter, Millicell inserted within a 24 well culture plate (NUNC, Melbourne, Australia). The inner well was maintained with 400 µL and the outer well contained 600 µL of culture media (EMEM + 15% FBS + 1% (*v*/*v*) penicillin-streptomycin). Cells were grown to confluence and reached a stable TEER value (≥1500 Ω cm^2^) over 10 days which was established from the initial assay. Next, both wells (inner and outer) had their media removed, washed three times with PBS (pH 7.4) and replaced with antibiotic free media. 

The suspensions of 10 µL (1.0 × 10^9^ CFU mL, OD = 1 at 600 nm) of bacterial isolates were inoculated into corresponding wells and incubated at 28 °C. Samples (100 μL) were collected from the outer well following 15, 30, 60 and 120 min of incubation, serially diluted using PBS and spread onto GYM agar. The culture media taken from the outer well was replaced at each sampled time interval. Plates were incubated for 8 days at 28 °C, and colony growth was checked every 24 h. The translocating bacteria were calculated and presented as the mean ± SEM [65,66].

### 4.8. Detection of Nocardiae Specific Phage Susceptibility of the Isolates

The six phages previously isolated from marine environments located on the Sunshine Coast where foam was evident [17,18] and had been stored in the University of the Sunshine Coast-Microbial Library (USC-ML) were selected for this assay (Table 3). All of the phages were in the Siphoviridae morphology group [67] with broad activity spectra within the genus *Nocardia* (Table 4).

Phages were propagated in peptone-yeast extract calcium (PYCa) broth [68], and routinely seeded with their designated propagation hosts (PH) until~1.0 × 10^8^ plaque forming units/ml for each phage isolate was achieved. A composite sample of six different phages was prepared using equal volumes of each phages and the phage susceptibility of the isolates was determined using spot test assay as described by Thomas et al. [8]. Lysis zones were evaluated using the index described by Jonns et al. [69].

### 4.9. Statistical Analysis

The total number of isolates were presented in the format of a bar and pie chart according to beach location, season, and isolation technique used. All charts were constructed by GraphPad Prism statistical software package (version 8.4.2, Graphpad, San Diego, CA, USA).

For the adhesion and translocation assays data were presented as mean ± standard deviation (SD), percentage (%), mean and standard error of the mean (SE). Data were analysed using one-way Brown-Forsythe and Welch’s ANOVA and differences were deemed statistically significant if *p* < 0.05; The Brown-Forsythe and Welch’s ANOVA was employed due to the heterogeneity of group variance. The Post-Hoc analysis was performed by Dunnett’s T3 multiple comparisons test to identify where the difference amongst the means occurred (significant if *p* < 0.05). 

The translocation assay results were analysed using traditional ANOVA in conjunction with Tukey’s multiple comparisons test due to smaller numbers and meeting the requirements of equal variance amongst the groups. Comparison between the *Nocardia* and the control group was conducted by means of an unpaired *t*-test with Welch correction, and deemed significantly different if *p* < 0.05. The GraphPad Prism statistical software package (version 8.4.2) was used for all statistical analysis.

## 5. Conclusions

In conclusion, the results obtained from this study further increases our understanding of the occurrence, diversity and human pathogenic potential of *Nocardia* isolates obtained from foaming coastal marine waters in the Sunshine Coast region of Australia. From the human health point of view the presence of such isolates in the coastal foams may present a risk, especially swimmers or individuals with underlying immune conditions, thus identification of such nocardiae might lead to the development of preventative measures by the local authorities. In addition, as indicated by Porri et al. [70] sea foam is important for “retaining larvae of polychaetes, mussels and barnacles near to the shore” thus foam’s unpolluted condition might be of significance for the health of near shore marine life as well.

## Figures and Tables

**Table 1:** Susceptibility of nocardiae isolates (*n* = 32) to various antibiotics.

Antibiotic	% Resistance
Ampicillin (10 μg)	15.7 (*n* = 5)
Ceftriaxone (30 μg)	75 (*n* = 24)
Cefotaxime (30 μg)	84.4 (*n* = 27)
Imipenem (10 μg)	6.3 (*n* = 2)
Amikacin (30 μg)	−
Minocycline (30 μg)	6.3 (*n* = 2)
Sulfamethoxazole/ trimethoprim (1.25/23.75 µg)	50 (*n* = 16) *
Erythromycin (15 μg)	6.3 (*n* = 2)
Tobramycin (10 μg)	63 (*n* = 20)

−: No resistance was detected, *: E-strip test for confirmation of the susceptibility against this antibiotic is required if the inhibition zone is <10 mm [31].

**Table 2:** *Nocardia* specific phage susceptibility of the isolates.

Strain Code	Phage Susceptibility
USC-21042, USC-21037	+++
USC-21044, USC-21021, USC-21046	++
USC-21018	±
USC-21034, USC-21050, USC-21049, USC-21035, USC-21030, USC-21025, USC-21006, USC-21038, USC-21039, USC-21036, USC-21048, USC-21017, USC-21028, USC-21027, USC-21010, USC-21029, USC-21047, USC-21026, USC-21022, USC-21040, USC-21043, USC-21043, USC-21016, USC-21012, USC-21011, USC-21024	−

+++: Highly susceptible (complete lysis), ++: Susceptible (complete partial lysis), +: Moderately susceptible (lysis and single plaques), ±: Low susceptibility (lysis but regrowth of the host), −: Not susceptible.

**Table 3:** Details of the *Nocardia*-specific phages.

Phage Codes	Propagation Hosts Used to Isolate the Phages
Ø1	*Nocardia soli* (DSMZ-44490)
Ø2	*Nocardia soli* (DSMZ-44490)
Ø3	*Nocardia soli* (DSMZ-44490)
Ø4	*Nocardia asteroides* (ACM-2963) *
Ø5	*Nocardia soli* (DSMZ-44490)
Ø6	*Nocardia asteroids* (ACM-2963) *

* ACM: Australian Collection of Microorganisms.

**Table 4:** Susceptibility of the *Nocardia* type strains to the composite phage suspension containing all six phages.

Type Strain IDs	Phage Susceptibility
*N. shimofusensis* (DSMZ 44733)	−
*N. takedensis* (DSMZ 44802)	±
*N. cumidelens/soli* (DSMZ 44488)	++
*N. soli* (DSMZ 44490)	+++
*N. uniformis* (DSMZ 43136)	++
*N. salmonicida* (DSMZ 40472)	++
*N. pseudovaccinii* (DSMZ 43406)	++
*N. veterana* (DSMZ 44445)	++
*N. fluminea* (DSMZ 44489)	−
*N. flavorosea* (DSMZ 44480)	+++
*N. asteroides* (ACM 131)	++
*N. asteroides* (ACM 2963)	++

+++: Highly susceptible (complete lysis), ++: Susceptible (complete partial lysis), +: Moderately susceptible (lysis and single plaques), ±: Low susceptibility, −: Not susceptible.

## Data Availability

Data is provided in the supplementary tables of the article.

## Supplementary Materials

The following are available online at https://www.mdpi.com/article/10.3390/pathogens10050579/s1. Table S1: Details of the *Nocardia* isolates; **^a^** Closest relative based on 16s rRNA gene sequence similarity. **^b^** +++: Highly susceptible (complete lysis), ++: Susceptible (complete partial lysis), +: Moderately susceptible (lysis and single plaques), ±: Low susceptibility (Lysis but regrowth of the host), −: Not susceptible. **^c^** Marine/beach locations situated on the Sunshine Coast, QLD, Australia; Table S2: S, susceptible. I, Intermediate. R, resistant. Footnote: S, susceptible. I, Intermediate. R, resistant, ¥: inhibition zone is <10 mm for Trimethoprim/sulphamethoxazole (1.25/23.75 µg) and an E-test strip is required to be subsequently performed as per Lebeaux, D., Bergeron, E., Berthet, J., Djadi-Prat, J., Mouniee, D., Boiron, P., Lortholary, O. and Rodriguez-Nava, V., 2019. Antibiotic susceptibility testing and species identification of *Nocardia* isolates: a retrospective analysis of data from a French expert laboratory, 2010–2015. *Clinical Microbiology and Infection*, *25*(4), 489–495.); Table S3: Differences in the adhesion capability of the 32 *Nocardia* isolates and control strains (HMLN-1 and 73–89) evaluated by microscopic method; Footnote: data shown are from triplicate experiments. Difference in the degrees of adhesion was found to be significant (*p* < 0.0001); Figure S1: Change in TEER values of calu-3 cells following 16 days post seeding. Data shown are from triplicate experiments and plotted as the mean ± SEM. Maximum TEER value (1588 Ω cm^2^) obtained on day 10 post seeding.
