# Supplementary material for: Isolation and Characterization of Nocardiae Associated with Foaming Coastal Marine Waters
Source: Pathogens. 2021 May 10;10(5):579. doi: 10.3390/pathogens10050579 (PMC8151412; doi:10.3390/pathogens10050579)
Supplement: Supplementary file 1 [file pathogens-10-00579-s001.zip › pathogens-1129980-supplementary.pdf]

Table S1. Details of the *Nocardia* isolates.

Local Isolates/ID	Closest Relative[a]	Percent Identity (%)	Phage Susceptibility[b]	Collection Location[c]	Month of Collection
**USC-21006**	MH261196.1 *Nocardia nova* strain MGA115	98.44%	−	Mooloolaba	February
**USC-21010**	MH261196.1 *Nocardia nova* strain MGA115	99.78%	−	Mooloolaba	February
**USC-21011**	MH261196.1 *Nocardia nova* strain MGA115	99.70%	−	Mooloolaba	February
**USC-21012**	MH261196.1 *Nocardia nova* strain MGA115	99.71%	−	Mooloolaba	February
**USC-21016**	MH261196.1 *Nocardia nova* strain MGA115	99.71%	−	Cotton Tree	March
**USC-21017**	MH261196.1 *Nocardia nova* strain MGA115	98.43%	−	Cotton Tree	March
**USC-21018**	MH261196.1 *Nocardia nova* strain MGA115	99.57%	±	Cotton Tree	March
**USC-21021**	KJ571085.1 *Nocardia grenadensis* strain T7-8	97.73%	++	Cotton Tree	March
**USC-21022**	MH261196.1 *Nocardia nova* strain MGA115	97.78%	−	Cotton Tree	March
**USC-21024** DSM 111829*	MH261196.1 *Nocardia nova* strain MGA115	98.97%	−	Mooloolaba	February
**USC-21025**	MH261196.1 *Nocardia nova* strain MGA115	99.42%	−	Mooloolaba	February
**USC-21026**	MH261196.1 *Nocardia nova* strain MGA115	99.71%	−	Mooloolaba	February
**USC-21027**	MH261196.1 *Nocardia nova* strain MGA115	99.78%	−	Mooloolaba	February
**USC-21028**	MH261196.1 *Nocardia nova* strain MGA115	99.63%	−	Mooloolaba	February
**USC-21029**	MH261196.1 *Nocardia nova* strain MGA115	99.06%	−	Mooloolaba	February
**USC-21030**	MH261196.1 *Nocardia nova* strain MGA115	98.21%	−	Cotton Tree	March
**USC-21032**	MH261196.1 *Nocardia nova* strain MGA115	98.49%	−	Cotton Tree	March
**USC-21034**	MH261196.1 *Nocardia nova* strain MGA115	98.97%	−	Mooloolaba	February
**USC-21035**	MH261196.1 *Nocardia nova* strain MGA115	99.56%	−	Mooloolaba	March
**USC-21036**	MH261196.1 *Nocardia nova* strain MGA115	99.64%	−	Mooloolaba	February
**USC-21037**	KY454539.1 *Nocardia testacea* strain F-161197	98.15%	+++	Cotton Tree	March
**USC-21038**	MH261196.1 *Nocardia nova* strain MGA115	99.64%	−	Mooloolaba	February
**USC-21039** DSM 111830*	KM978823.1 *Nocardia higoensis* strain A6	97.01%	−	Mooloolaba	February
**USC-21040**	MH261196.1 *Nocardia nova* strain MGA115	97.14%	−	Mooloolaba	October

**USC-21042**	NR_149226.1 *Nocardia rayongensis* strain RY45-3	96.79%	+++	Maroochydore	October
**USC-21043**	HM584914.1 *Nocardia nova* strain CBU 09/875	97.99%	−	Maroochydore	October
**USC-21044** DSM 111630*	NR_117402 *Nocardia niigatensis* strain W8186	97.84%	++	Maroochydore	October
**USC-21046** DSM 111727*	NR_109056.1 *Nocardia goodfellowii* strain A2012	93.03%	++	Maroochydore	October
**USC-21047**	MK302233.1 *Nocardia tengchongensis* strain JBRI-MO-0018	97.19%	−	Maroochydore	October
**USC-21048** DSM 111726*	NR_117320.1 *Nocardia elegans*	96.50%	−	Maroochydore	October
**USC-21049**	MN620411.1 *Nocardia sp.* strain 19D1V24	97.26%	−	Maroochydore	October
**USC-21050**	NR_117322.1 *Nocardia flavorosea*	96.80%	−	Maroochydore	October

[a]Closest relative based on 16s rRNA gene sequence similarity. [b] +++: Highly susceptible (complete lysis), ++: Susceptible (complete partial lysis), +: Moderately susceptible (lysis and single plaques), ±: Low susceptibility (Lysis but regrowth of the host), −: Not susceptible. [c] Marine/beach locations situated on the Sunshine Coast, QLD, Australia. *:Leibniz Institute DSMZ – German Collection of Microorganisms and Cell Cultures (DSMZ) strain numbers for 5 deposited isolates which are publicly available.

Table S2. Antibiotic susceptibility testing results.

STRAIN ID	ZONE DIAMETER (mm) /ANTIBIOTIC SUSCEPTIBILITY<br>*Interpretation threshold (mm) as described by Lebeaux, D., Bergeron, E., Berthet, J., Djadi-Prat, J., Mouniee, D., Boiron, P., Lortholary, O. and Rodriguez-Nava, V., 2019. Antibiotic susceptibility testing and species identification of *Nocardia* isolates: a retrospective analysis of data from a French expert laboratory, 2010–2015. *Clinical Microbiology and Infection*, *25*(4), 489–495.							
	Ampicillin (10μg), ≥21 (S)*, <16 (R)*	Imipemen (10μg), ≥24 (S)*, <17 (R)*	Cefotaxime (30μg), ≥26 (S)*, <23 (R)*	Cetfriaxone (30μg), ≥26 (S)*, <23 (R)*	Tobramycin (10μg), ≥18 (S)*, <16 (R)*	Amikacin (30μg), ≥17 (S)*, <15 (R)*	Minocycline (30μg), (30IU, ≥26 (S)*, <23 (R)*	Erythromycin (15μg), 15IU, ≥22 (S)*, <17 (R)*	Trimethoprim/sulphamethoxazole¥ (1.25/23.75 μg) ≥16 (S)*, <10 (R)*
**USC-21006**	28 (S)	60 (S)	0 (R)	10 (R)	12 (R)	58 (S)	30 (S)	40 (S)	20 (S)
**USC-21010**	34 (S)	68 (S)	0 (R)	0 (R)	12 (R)	60 (S)	0 (R)	44 (S)	0 (R)¥
**USC-21011**	30 (S)	60 (S)	0 (R)	10 (R)	14 (R)	60 (S)	52 (S)	48 (S)	0 (R)¥
**USC-21012**	32 (S)	60 (S)	0 (R)	12 (R)	14 (R)	50 (S)	42 (S)	42 (S)	0 (R)¥
**USC-21016**	22 (S)	56 (S)	0 (R)	0 (R)	10 (R)	40 (S)	22 (S)	46 (S)	28 (S)
**USC-21017**	26 (S)	56 (S)	8 (R)	0 (R)	17 (I)	54 (S)	30 (S)	44 (S)	40 (S)
**USC-21018**	36 (S)	54 (S)	16 (R)	0 (R)	10 (R)	24 (S)	32 (S)	42 (S)	30 (S)
**USC-21021**	30 (S)	42 (S)	34 (S)	38 (S)	44 (S)	44 (S)	46 (S)	20 (I)	50 (S)
**USC-21022**	24 (S)	54 (S)	0 (R)	0 (R)	14 (R)	39 (S)	34 (S)	40 (S)	36 (S)
**USC-21024**	32 (S)	60 (S)	0 (R)	0 (R)	16 (I)	62 (S)	50 (S)	40 (S)	0 (R)¥
**USC-21025**	28 (S)	60 (S)	0 (R)	0 (R)	0 (R)	66 (S)	30 (S)	40 (S)	0 (R)¥
**USC-21026**	14 (R)	52 (S)	0 (R)	0 (R)	0 (R)	58 (S)	26 (S)	46 (S)	0 (R)¥
**USC-21027**	32 (S)	62 (S)	0 (R)	0 (R)	8 (R)	60 (S)	30 (S)	44 (S)	0 (R)¥
**USC-21028**	20 (I)	52 (S)	0 (R)	0 (R)	10 (R)	58 (S)	28 (S)	46 (S)	0 (R)¥
**USC-21029**	20 (I)	64 (S)	0 (R)	12 (R)	10 (R)	56 (S)	24 (I)	50 (S)	0 (R)¥
**USC-21030**	34 (S)	68 (S)	0 (R)	50 (S)	8 (R)	42 (S)	26 (S)	48 (S)	0 (R)¥
**USC-21032**	20 (I)	64 (S)	0 (R)	20 (R)	12 (R)	50 (S)	30 (S)	40 (S)	20 (S)
**USC-21034**	16 (I)	56 (S)	0 (R)	0 (R)	14 (R)	48 (S)	30 (S)	28 (S)	0 (R)¥
**USC-21035**	30 (S)	70 (S)	0 (R)	0 (R)	14 (R)	50 (S)	36 (S)	52 (S)	0 (R)¥
**USC-21036**	16 (I)	42 (S)	0 (R)	0 (R)	34 (S)	32 (S)	36 (S)	44 (S)	40 (S)

**USC-21037**	28 (S)	50 (S)	36 (S)	32 (S)	60 (S)	60 (S)	30 (S)	10 (R)	60 (S)
**USC-21038**	26 (S)	60 (S)	0 (R)	0 (R)	8 (R)	52 (S)	32 (S)	46 (S)	0 (R)¥
**USC-21039**	32 (S)	42 (S)	22 (R)	30 (S)	32 (S)	40 (S)	42 (S)	16 (R)	42 (S)
**USC-21040**	24 (S)	42 (S)	0 (R)	20 (R)	14 (R)	40 (S)	20 (R)	50 (S)	30 (S)
**USC-21042**	62 (S)	64 (S)	40 (S)	39 (S)	62 (S)	70 (S)	60 (S)	50 (S)	30 (S)
**USC-21043**	10 (R)	48 (S)	0 (R)	0 (R)	0 (R)	60 (S)	30 (S)	40 (S)	0 (R)¥
**USC-21044**	0 (R)	0 (R)	0 (R)	0 (R)	38 (S)	40 (S)	30 (S)	24 (S)	34 (S)
**USC-21046**	24 (S)	42 (S)	30 (S)	32 (S)	50 (S)	48 (S)	40 (S)	48 (S)	40 (S)
**USC-21047**	0 (R)	0 (R)	0 (R)	0 (R)	40 (S)	38 (S)	38 (S)	40 (S)	38 (S)
**USC-21048**	24 (S)	56 (S)	10 (R)	26 (S)	40 (S)	50 (S)	40 (S)	28 (S)	0 (R)¥
**USC-21049**	20 (I)	64 (S)	0 (R)	0 (R)	14 (R)	44 (S)	30 (S)	34 (S)	0 (R)¥
**USC-21050**	14 (R)	46 (S)	32 (S)	39 (S)	34 (S)	56 (S)	48 (S)	24 (S)	44 (S)

S, susceptible. I, Intermediate. R, resistant. Footnote: S, susceptible. I, Intermediate. R, resistant, ¥: inhibition zone is <10mm for Trimethoprim/sulphamethoxazole (1.25/23.75 µg) and an E-test strip is required to be subsequently performed as per Lebeaux, D., Bergeron, E., Berthet, J., Djadi-Prat, J., Mouniee, D., Boiron, P., Lortholary, O. and Rodriguez-Nava, V., 2019. Antibiotic susceptibility testing and species identification of *Nocardia* isolates: a retrospective analysis of data from a French expert laboratory, 2010–2015. *Clinical Microbiology and Infection*, *25*(4), 489–495.).

pathogens

Table S3. Differences in the adhesion capability of the 32 *Nocardia* isolates and control strains (HMLN-1 and 73-89) evaluated by microscopic method.

Isolates & Strains	Average No. of Adhering Bacteria/Cell[a]	Average No. of Adhering Bacteria /Cell[a]	Percentages of Cells Showing Adhering Bacteria[b]	Adherence Pattern Phenotype[c]
**USC-21006**	4.3 ± 0.3	4.3 ± 2.9	22.0	DA
**USC-21010**	1.4 ± 0.4	1.4 ± 3.4	5.0	DA
**USC-21011**	0.1 ± 0.0	0.1 ± 0.4	0.7	DA
**USC-21012**	0.1 ± 0.0	0.1 ± 0.3	0.7	DA
**USC-21016**	0.2 ± 0.1	0.2 ± 0.7	1.3	DA
**USC-21017**	4.3 ± 0.8	4.3 ± 7.2	9.3	DA
**USC-21018**	3.4 ± 0.4	3.4 ± 3.7	15.0	DA
**USC-21021**	5.3 ± 0.6	5.3 ± 5.6	15.3	DA
**USC-21022**	0.3 ± 0.1	0.3 ± 0.9	2.3	DA
**USC-21024**	0.1± 0.1	0.1 ± 0.6	0.3	DA
**USC-21025**	10.8 ± 0.8	10.8 ± 6.5	31.0	LA
**USC-21026**	0.5 ± 0.1	0.5 ± 1.2	4.3	DA
**USC-21027**	0.5 ± 0.1	0.5 ± 1.1	5.3	DA
**USC-21028**	1.3 ± 0.3	1.3 ± 3.0	5.3	DA
**USC-21029**	0.7 ± 0.2	0.7 ± 1.6	4.7	DA
**USC-21030**	8.9 ± 0.5	8.9 ± 4.0	52.7	DA
**USC-21032**	0.3 ± 0.1	0.3 ± 0.8	3.3	DA
**USC-21034**	15.5 ± 0.8	15.5 ± 6.6	99.3	DA
**USC-21035**	20.0 ± 0.1	20.0 ± 0.9	60.7	DA
**USC-21036**	6.5 ± 0.8	6.5 ± 6.6	16.3	LA
**USC-21037**	0.1 ± 0.1	0.1 ± 0.5	1.7	DA
**USC-21038**	16.5 ± 1.0	16.5 ± 9.0	21.7	DA
**USC-21039**	4.6 ± 0.5	4.6 ± 4.2	18.0	DA
**USC-21040**	0.3 ± 0.1	0.3 ± 1.1	1.7	DA
**USC-21042**	5.4 ± 0.6	5.4 ± 5.5	16.7	DA
**USC-21043**	0.2 ± 0.1	0.2 ± 0.8	1.3	DA
**USC-21044**	16.9 ± 0.7	16.9 ± 6.2	88.7	DA
**USC-21046**	4.7 ± 0.6	4.7 ± 5.0	15.0	DA
**USC-21047**	0.5 ± 0.2	0.5 ± 1.3	4.7	DA
**USC-21048**	5.0 ± 0.6	5.0 ± 5.3	16.0	DA
**USC-21049**	16.5 ± 1.0	16.5 ± 9.0	94.7	DA
**USC-21050**	19.4 ± 0.9	19.4 ± 7.7	98.3	DA
**HLMN-1**	21.8 ± 0.6	21.8 ± 5.1	90.0	DA
**73-89**	21.0 ± 0.4	21.0 ± 3.4	85.7	DA

Data shown are from triplicate experiments. Difference in the degrees of adhesion was found to be significant ($P < 0.0001$) [a] Bacterial adhesion determined by enumerating the number of bacterial cells per 25 randomly selected Calu-3 cells in triplicates. [b] Mean Percentage calculated by identifying bacterial cells displaying adherence to 100 randomly selected Calu-3 cells.[c] DA: diffuse; LA: Localized.

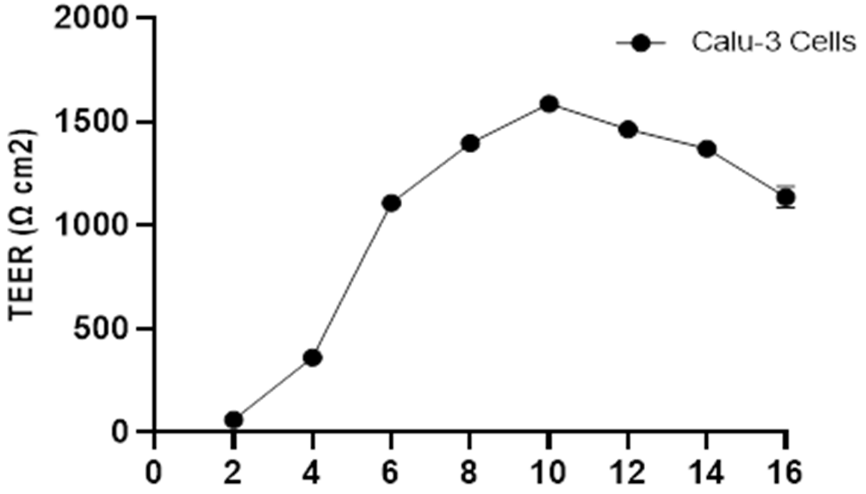

Figure S1. Change in TEER values of calu-3 cells following 16 days post seeding. Data shown are from triplicate experiments and plotted as the mean ± SEM. Maximum TEER value (1588 Ω cm^2) obtained on day 10 post seeding.
